# Implications of holistic face processing in autism and schizophrenia

**DOI:** 10.3389/fpsyg.2013.00414

**Published:** 2013-07-05

**Authors:** Tamara L. Watson

**Affiliations:** School of Social Science and Psychology, University of Western SydneySydney, NSW, Australia

**Keywords:** vision, face recognition, autism, schizophrenia, holistic coding, configurational coding

## Abstract

People with autism and schizophrenia have been shown to have a local bias in sensory processing and face recognition difficulties. A global or holistic processing strategy is known to be important when recognizing faces. Studies investigating face recognition in these populations are reviewed and show that holistic processing is employed despite lower overall performance in the tasks used. This implies that holistic processing is necessary but not sufficient for optimal face recognition and new avenues for research into face recognition based on network models of autism and schizophrenia are proposed.

To those who are able to recognize the information displayed on a face it can be almost impossible to imagine what it would be like to be unable to do so. This perception is largely effortless and automatic, yet there are many people who have considerable impairment in face recognition. Even beyond those with prosopagnosia, or “face blindness,” there are populations of people whose perception of faces has been shown to be impaired to the extent that it interferes with their social functioning. The question of the cause of impairment will often come back to the suggestion that they lack the ability to make use of the global, holistic or configurational information that has been shown to be of crucial importance in successful face perception. The research carried out to address this question is very informative, not only in aiding our understanding of the face recognition deficits directly, but also of how face recognition occurs in those without a deficit.

Two populations of people that have been studied are those with autism or schizophrenia. Both groups have been shown to have a characteristic local processing bias (Phillips and Silverstein, 2003; Happé and Frith, 2006) making both populations natural candidates for a lack of configurational or holistic face processing (Frith et al., 1983; Tantam et al., 1989; Schwartz et al., 2002). Comparing and contrasting the results of studies in these two population groups can therefore provide particular insight into the nature of configurational processing. In other words, by studying the face recognition abilities of two groups of people who are thought to have a bias against the key visual processing strategy needed for the successful interpretation of faces we should be able to understand something fundamental about how normal populations recognize faces.

## Holistic encoding of faces

Before discussing face recognition studies carried out with these populations, holistic encoding of faces should be addressed. As stated above, there is an almost universal agreement amongst researchers in this area that faces are not perceived as an additive accumulation of evidence from the explicitly nameable parts of a face alone. Rather than a “part-based” processing strategy, a more holistic or configurational processing strategy is applied (for reviews see Maurer et al., 2002; Piepers and Robbins, 2012). There are several conceptualizations of holistic or configurational processing. Holistic processing refers to the face being represented within the visual system as an indivisible whole (Bradshaw and Wallace, 1971; Tanaka and Farah, 1993), where each face is essentially perceived as a match to a face template. Configurational processing broadly refers to the importance of the relationships between parts in the processing strategy. The several models of configurational processing adhere generally to the idea of a hierarchy of relational properties within the face (Diamond and Carey, 1986; Rhodes, 1988; Leder and Bruce, 2000; Bartlett et al., 2003). Diamond and Carey (1986) have proposed that first order configurational relations refer to the gross placement of features within the face outline, such that two horizontally separated eyes sit above a centrally placed nose which in turn sit above the centrally positioned mouth. Second order configurational properties refer to the finer scale relations between parts of the face and might include information, such as the distance between the eyes. There is general agreement that faces are a special type of visual stimulus for humans (see McKone and Robbins, 2011). The unique importance of faces may be realized through a specialized system devoted genetically to analyse faces (Morton and Johnson, 1991), or due to the extreme expertise developed as a result of paying such close attention to faces throughout our lifespan (Diamond and Carey, 1986). One key aspect of the “specialness” is that the faces we regularly interact with are recognized via a holistic or configurational type of processing strategy within the brain, setting them apart from most other visual objects (see McKone and Robbins, 2011). There is less overall agreement between researchers on the details of how holistic or configurational processing is achieved in all the circumstances in which faces are viewed or what role these strategies play in all the tasks for which faces are a primary source of information. Investigating populations with altered face recognition abilities should be beneficial in adding to the discussion of what successful face recognition requires and what holistic/configurational processing entails.

## Autism and schizophrenia

People with autism and schizophrenia are of interest when considering holistic/configurational face processing because both groups have been proposed to have a local or featural processing bias (Dakin and Frith, 2005; Happé and Frith, 2006) or a fragmented sensory processing style (Cutting, 1989; Phillips and Silverstein, 2003; Butler et al., 2008a). This entails a focus on localized, isolated aspects of a scene or details that others overlook. Both groups also suffer from poor social skills that have been proposed to at least partly arise from poor face recognition skills (Langdell, 1978; Couture et al., 2006). Given this context it is natural to enquire whether poor face recognition performance arises from the local processing bias and hence a lack of holistic/configurational face processing. It is of interest to note contemporary debate concerning the relationship between autism and schizophrenia as disorders. Opposing views are being explored, suggesting that schizophrenia and autism are either unrelated, manifestations of the same underlying cause or that they are opposing ends of a shared spectrum (for a discussion of this see Crespi et al., 2010). The similarity that the shared suggestion of a local processing bias implies is an intriguing idea. Whether the two diagnoses ultimately share a common neural basis or not, exploring the results of studies on the currently defined populations remains very informative when it comes to understanding the holistic nature of face recognition.

Autism and Autism Spectrum Disorders (ASD) have been characterized by impairments in communication, social cognition and repetitive behaviors according to the DSM-IV-TR (American Psychiatric Association, 2000)^1^. Also crucial to the presentation of autism is a preoccupation with details or parts of objects. This is an aspect that has been recognized very early as a key feature of the diagnosis (Kanner, 1943). The onset of noticeable symptoms occurs in early childhood and as such it is a developmental disorder. Schizophrenia, on the other hand, most commonly onsets in late adolescence and is characterized by a diverse range of symptoms that include delusions, hallucinations, blunted affect, disorganized communication and reduced motivation (American Psychiatric Association, 2000). It is also recognized that people with schizophrenia have poorer performance on cognitive tests and reduced social functioning (Marwick and Hall, 2008). Although a preoccupation with details is not a central characteristic of the disorder, as it is in autism, there is ample evidence to suggest that perceptually and perhaps also cognitively, people with schizophrenia can find global and contextual processing quite difficult as localized features gain prominence in their consciousness (Braff, 1993; Butler et al., 2008a). As testament to the similarity in the local/global processing relationship in autism and schizophrenia there are several theoretical proposals/models in the scientific literature on both disorders that are almost interchangeable, or at least complement each other very well (e.g., Mottron and Burack, 2001; Phillips and Silverstein, 2003; Happé and Frith, 2006; Krishnan et al., 2011). The weak central coherence theory of autism (Happé and Frith, 2006) and the hierarchical temporal processing deficit model of psychosis (Krishnan et al., 2011) are examples of a theory and a model that could be addressing the exact same issue.

## Local bias in autism

**Figure 1:**
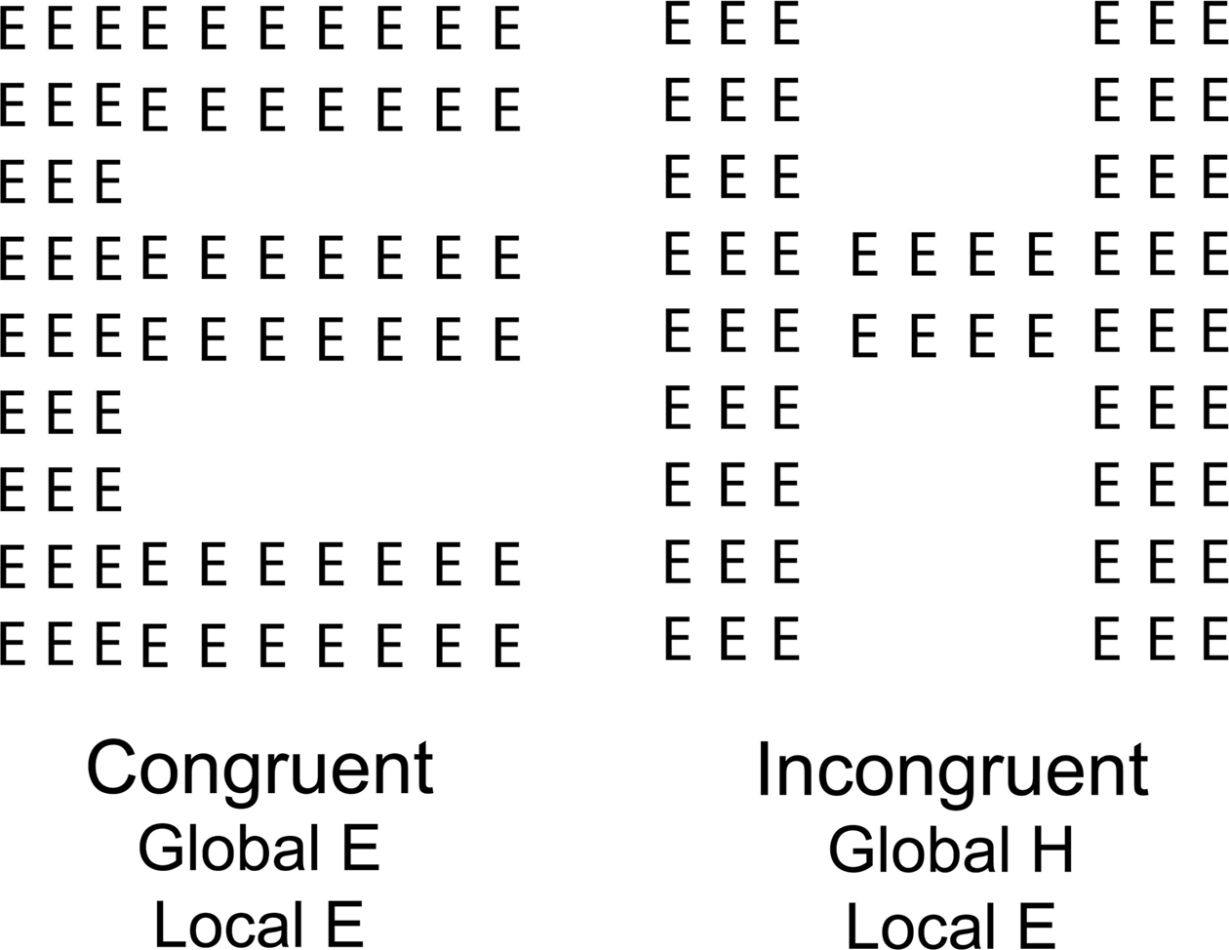
**An example of a Navon task stimulus (Navon, 1977).** The participant is asked to identify a target letter that can be present at both the local and global level (congruent stimulus) or may be present at either the local or global level (incongruent stimulus).

The weak central coherence theory originally posited that people with autism were impaired at global processing (Happé, 1996; Frith, 2003). With experimental evidence it has been modified to propose that rather than being a true deficit, global processing can still be achieved but generally there is an automatic bias toward local processing (Happé and Frith, 2006). This theory is accompanied by several neural models that include reduced connectivity throughout the brain caused by a reduction in synchronization of activity (Brock et al., 2002), or a reduction in feedback connectivity causing a failure of top-down connectivity (Frith, 2004). Each model proposes a different mechanism that could be differentiated via the detail, however, the broader implications are the same. Tasks requiring a decision to be made about localized elements of a stimulus will be easier or more accurate than tasks requiring a global view. Supporting this theory are data collected using a Navon task requiring identification of a letter that could be presented at a local or a global level within the stimulus (Navon, 1977). Navon letters are constructed such that many small letters (local stimuli) are arranged to form a larger letter (the global stimulus) (See Figure 1). The global and the local letters can correspond (an E made from many smaller Es) or oppose (a larger H made from many small Es) each other. Autistic children show reduced accuracy (Plaisted et al., 1999) and slower reaction time (Behrmann et al., 2006) identifying the global letter when it is incongruent with the local letter when compared to identifying the local letter in the opposite stimulus configuration, or when both the local and global letter match. These results are suggestive of a local processing superiority and are opposite to the results found in a control population. This version of the task requires divided attention between both the local and the global level of the stimulus as the children need to identify whether a target letter was present at either level of the stimulus. In more directly assessing whether the children were unable to use a global processing strategy, when directed to selectively attend to either the local or the global level of the Navon stimulus, autistic children performed as the control children did (Plaisted et al., 1999). The local advantage was not present. These results suggest that children may show a local processing bias when faced with complex stimuli. When directed as to what level of the stimulus is important, however, the children are able to engage a global strategy to successfully gain a global advantage and produce a global interference. This suggests that global processing is not impossible, however, it may not be preferentially or automatically engaged.

Evidence from the Navon task is supportive of a weak central coherence, however, it also clearly shows the difficulty in pinning down the cause of a local processing bias. The extent to which we can make a strong claim for a particular cause for a local bias rests very heavily on the detail of the task the participant is carrying out. Further evidence using a range of other tasks also comes out in general support of the idea of a local processing bias, to the extent that the task can be assumed to allow for this. The Rey-Osterrieth Figure drawing (Osterrieth, 1944) and the block design task (Kohs, 1923; Wechsler, 1974, 1981) are two examples of other tasks aimed at investigating local bias. The block design task requires participants to recreate a compound shape by combining individual square blocks with component shapes on the surface. This task requires that a participant break up the global target shape into smaller units and match them to the shapes available on the blocks. In many instances participants with autism or ASD are found to be more accurate or faster at combining the individual blocks into the target shapes (Ohta, 1987; Bowler, 1992; Shah and Frith, 1993). One particular study by Shah and Frith (1993) found that participants with autism only outperformed controls on aspects of the task that required the segmentation of wholes into parts while other spatial abilities that contributed to the task, such as mental rotation, were not enhanced. On the other hand the Rey-Osterrieth figure drawing task requires participants to copy a complex abstract figure composed of small geometric elements (Osterrieth, 1944). It is thought that this task can be approached via a local or global strategy. Either placing each local element in position serially, or outlining the global features and then filling in local details. Researchers have looked for signs of the strategy used by participants by counting the number of elements drawn or whether people begin with the global outline or local elements first (Jolliffe and Baron-Cohen, 1997; Kuschner et al., 2009). Studies using this approach find inconclusive results that really reflect the limited ability of the task to tap into a local or global strategy. Kuschner et al. (2009) suggests that the high functioning participants in their study might view both the more “global” and more “local” elements as equivalent, a sensible suggestion given the geometric nature of the figure. Making the Rey-Osterrieth figure a poor tool for assessing local bias. One similar test assessed the copying of impossible figures (Mottron et al., 1999). In this case participants with autism were more accurate as they appeared to be less impeded by the impossible nature of the image as a whole (Brosnan et al., 2004). Behrmann et al. (2006) mentioned above using the Navon task, also carried out a range of local/ global tasks including a priming task that showed people with autism did not group elements to benefit from a global similarity to a primed shape. They found that the same individuals were much slower at making a same/different face discrimination task but did not show different spatial frequency thresholds. They conclude that people with autism may be able to derive a global percept with effort but generally demonstrate a bias toward local information. Despite the difficulty in creating a task that faithfully tests for a local bias and the heterogeneity of the population of people with autism the literature supports the claim that this population displays a local processing bias and therefore could be expected to process faces in a less then holistic manner.

## Local bias in schizophrenia

Similar to autism, a form of local processing bias has been proposed to be evident in schizophrenia (Cutting, 1989; Phillips and Silverstein, 2003; Butler et al., 2008a). The hierarchical temporal processing deficit model of psychosis (Krishnan et al., 2011) proposes changes in the ability of the sensory system to maintain adequate temporal resolution and precision of neural responses result in an imperfect flow of information throughout the sensory system of those with schizophrenia. It is proposed that the ability of the sensory system to match up incoming visual data to stored representations of a stimulus is degraded. This leads to impairment in the ability to create sufficiently invariant representations of stimuli, resulting in an inability of the higher levels of the sensory hierarchy to inform and shape the expectations of the lower levels. This model also proposes that global/holistic processing can occur. It suggests that on balance, however, with imperfect ability to match prior expectation to incoming data, the stimulus will be processed in a more part-based manner and any holistic processing that does occur will be altered compared to a control population.

To investigate global processing the Navon task (Navon, 1977) has also been used in studies of people with schizophrenia. One study using the divided attention version of the Navon local-global task, where a target letter may be presented at either the local or global level, have shown that patients have a local bias. They have slower reaction times and make more errors when identifying a global letter comprised of smaller distracter letters compared to control participants who were found to be equally fast and accurate at identifying target letters at either the local or global level (Johnson et al., 2005). A previous study using the divided attention task in this population, however, have found a local deficit (Granholm et al., 1999). The authors of this result themselves suggest it may be due to differences in the salience of the stimuli at different levels and therefore driven by a more complex relationship between the stimulus and attention. This is supported by studies investigating shifts of selective attention that show a problem only when attention is instructed to shift from a global to a local level between subsequent trials (Coleman et al., 2009). The suggestion from these results would be that both local and global processing are possible, however, the interaction between the two is altered leading to a local processing bias under divided attention circumstances.

A range of results from other similar tasks also point to the same conclusion. Silverstein et al. (1996) used a task similar to the Navon task, however, the distracter characters were grouped in such a way that they were more or less distracting when identifying a target letter in a crowded array. In this task, those with poor pre-morbid functioning were less affected by greater grouping of distracters. Similarly, using symmetrical stimuli for which the symmetry could either support or disrupt performance Knight et al. (2000) found that patients with poor pre-morbid functioning were inhibited by symmetry when it was a disruptive cue, just as controls were. Unlike controls, they were not facilitated by symmetry when it was useful. Knight et al. (2000) suggests that the use of symmetry to enhance performance appeared stronger in controls who had more experience of the stimuli. Suggesting that the patient population may have engaged in some automatic global processing but not been able to switch strategy to make use of it when it was useful. Similar to this result (and unlike those found with participants with autism) people with schizophrenia are sometimes shown to be impaired in tasks where a local bias should facilitate performance. Using the block design task (Kohs, 1923; Wechsler, 1974, 1981), Zhai et al. (2011) have shown that patients are impaired compared to controls. In this same study the patients were also shown to be impaired at a mental rotation task. Shah and Frith (1993) found a dissociation between these types of processing in an autistic population, therefore these results really highlight the difficulty in teasing apart a local bias from a more general visuo-spatial deficit in schizophrenia. Also unlike the results found in autism, Seidman et al. (2003) have shown that participants with schizophrenia are less accurate and at the same time they do use a more detail oriented copy and recall style when completing the Rey-Osterrieth figure drawing task (Osterrieth, 1944). On balance, in comparing the overall pattern of the results within schizophrenia, an underlying local processing bias or less efficient use of a global strategy appear to be a key problem that is accompanied by a broader range of visuo-spatial and cognitive problems than are apparent in autism.

Results such as these inform the theories and models of visual processing in schizophrenia and autism alike by highlighting that a form of local bias, rather than a global deficit, is apparent in both disorders. At times, it seems that the global processing can be achieved. However, as the theories suggest, a global strategy appears to need to be prompted. It is a reasonable question therefore, to ask whether these populations automatically apply a configurational/holistic processing style to faces and whether they can do this successfully.

## Face processing in autism

There are many kinds of information that can be gained from successful visual face processing. For example, faces can help us identify previously encountered individuals, they can signal age, display emotion and much more. Configurational/holistic face processing may be differentially important depending on the facial information relevant for the task at hand, however, it is generally considered to be very important for individuating faces. There are many tasks designed to assess the application of configurational/holistic face processing in identity judgments spanning the range of those that require none to very little long-term memory, those that require significant familiarity with the test faces, those that manipulate the race of the stimuli used and many more. In this paper I will be addressing only a subset of these tasks, looking at the most basic application of configurational/holistic processing with very little memory requirement, using unfamiliar face stimuli. Although this may seem a restricted set of evidence, it encompasses the foundational tasks used to assess basic holistic/configurational processing and therefore provides a valuable contribution. In particular, tasks requiring more than an insubstantial memory component have been specifically excluded. Memory has been shown to play a significant role in the face recognition abilities of both autistic (see Weigelt et al., 2012) and schizophrenic individuals (see Marwick and Hall, 2008). As holistic face processing and not memory encoding is the focus of this paper these results will not be explored. I will look at the evidence generated from a basic set of face processing tasks carried out by participants with autism and schizophrenia in turn.

The face inversion effect is one method of investigating configurational processing. In general participants are much worse at recognizing an upside down than an upright face when compared to other visual objects (Yin, 1969). It is proposed that we do not have a configurational/holistic template for a face in the inverted orientation and therefore fall back on a part-based approach with these stimuli (Young et al., 1987; Farah et al., 1995). It stands to reason therefore that children with autism, if relying on a more part-based approach than typically developing children, may not exhibit an inversion effect. Results with this task are mixed. Hobson et al. (1988) found that children with autism have a smaller face inversion effect suggesting less cofigurational/holistic processing. Tantam et al. (1989) have also found a reduced inversion effect when children were asked to find the odd face out. Here the children with autism showed equivalent performance to control children when presented with inverted faces but did not gain an advantage with upright faces. However, Scherf et al. (2008) have shown that children and adults with autism exhibit a face inversion effect that is comparable to control participants despite performance being worse overall. This result would suggest that cofigurational/holistic processing is occurring- if simply inverting the face is a true method of eliminating holistic/configurational processing. The inversion effect alone is an indirect measure of configurational processing, however, as we can currently only assume that a part-based strategy is the fall back position for visual processing of inverted faces. This is because configurational information remains intact so long as the viewer is able to rotate their face coordinate system.

**Figure 2:**
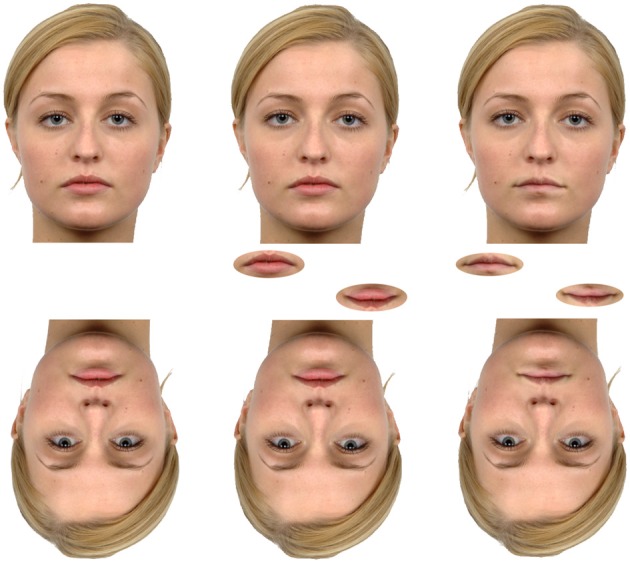
**Featural and configurational changes to upright and inverted faces.** The central face is the original (Langner et al., 2010) while the face to the left has eyes that have been moved closer to the bridge of the nose. The face to the right has had the mouth replaced with that of another person. Between the upright and inverted faces are the target features isolated from the face. Configurational/holistic processing suggests the changes should be more apparent in the upright, whole faces compared to the inverted faces or when the features are isolated.

A more direct measure is to create a circumstance under which configurational information is removed. In terms of face processing, a more direct measure of holistic processing was first assessed in children with autism by Joseph and Tanaka (2003). They used the part-whole task in which participants are generally found to be better at recognizing faces with changed featural components (different mouth for example) when presented in the context of a whole face at test (Figure 2). Joseph and Tanaka (2003) also assessed face part recognition ability in the context of upright or inverted faces. In this instance, a reliance on a part-based strategy by children with autism should be apparent by a lack of better performance when recognizing whole faces compared to parts and by a lack of an inversion effect. They should therefore show a different pattern of results across conditions compared to typically developing children. They found that normally developing children were better able to match a face that had been seen before when the feature that differed at test was presented in the context of a whole face compared to when presented alone on the screen (this is a whole-face advantage). A whole face inversion cost and an absence of a whole-face advantage was also found when stimuli were presented upside down. In comparison, children with autism were found to show similar performance to control children only when the mouth was the target feature changed at test. When the eyes were the target feature no upright whole face advantage was found. Wolf et al. (2008) also carried out similar tests on children with ASD and found a very similar result. Children in the ASD group were less accurate when the eyes were changed but equally accurate as the controls when the mouths were changed. In this study Wolf et al. (2008) also included a manipulation where the eyes or the mouth were simply masked out and participants were asked to match identity across a 45° change in viewpoint. In this case the ASD participants performed more poorly overall compared to the typically developing children, however, the pattern of results showed that the performance was not different between the groups depending on the feature that was blocked out. Another study by Faja et al. (2009) also found the whole-face advantage in an ASD group despite poorer overall performance. This study did not find a clear reliance on the mouth area as found in the previously discussed studies.

These complicated results would suggest that holistic processing is apparent but different in autistic children depending on the task. As opposed to the eye region being particularly important, they suggest the holistic representation constructed by children with autism seems to favor the mouth area. Depending on the task, however, this result can be modulated in favor of the those with autism displaying overall holistic processing but with generalized poor performance. This is exemplified in the results of another study that used the part-whole task. This time the participants were cued to attend to either local features or the whole face (López et al., 2004). This study found that high functioning adolescents experienced better performance with the whole face when cued to attend that way and no difference between the part or whole face conditions when uncued.

Another task aiming to tap into holistic processing in faces is quite similar to the part whole effect. Rather than switching out the features of the face, the original features can be manipulated by changing their shape (a featural change) or moving them within the context of the whole face (a change in configuration) (Tanaka and Sengco, 1997). For example, the eyes can be enlarged or they can be moved further apart (Figure 2). By asking participants to discriminate between instances of faces manipulated in this way the sensitivity to facial configuration can be assessed. Using this type of task Wolf et al. (2008) found results similar to those found by Joseph and Tanaka (2003) using the part whole task. Participants with ASD performed with the same pattern of results as did the control group except that the control group were better at identifying changes of both eye position and eye shape than the ASD participants. No difference in performance between groups was found when these changes were applied to the mouth. Faja et al. (2009) also carried out this task including only the position change condition, with a large or small position manipulation. They found that typically developing participants were simply more sensitive to the changes presented. Rutherford et al. (2007) have also tested the sensitivity of a group of participants with ASD to changes in the position of either the eyes or mouth within an upright or inverted face. In finding that many of the participants in the patient group were unable to meet their criteria for threshold performance when the position of the eyes was manipulated, yet were able to meet criteria when the mouth was manipulated, they investigated their results on an individual basis. Interestingly they found that their patient group clustered into two groups, one with almost no impairment and another with severe impairment in processing displacement of the eyes. Crucially, the group with impaired eye displacement discrimination still showed the same patterns of inversion effects as the controls, they were just less sensitive overall. A result that strongly suggests those in the patient population who were less able to discriminate faces based on eye position were none the less employing a configurational strategy to carry out the task.

**Figure 3:**
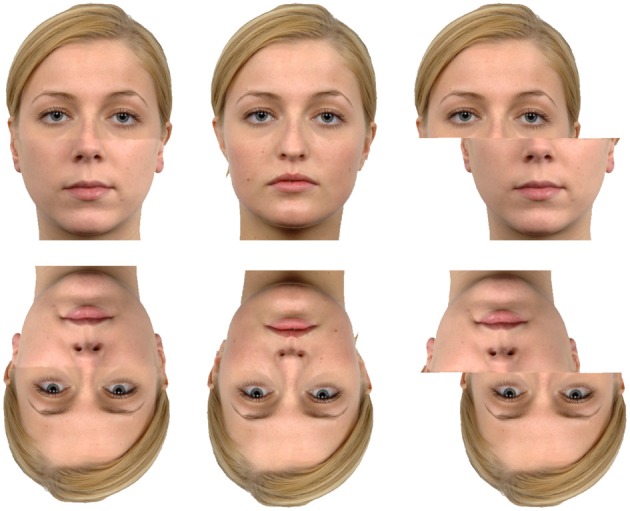
**The Composite effect.** The central face is the original (Langner et al., 2010) while the left and right faces are composites of two identities. The identity of one half of the face is easier to match when the two halves of the face are misaligned (**Right**) compared to aligned (**Left**). This effect is reduced when the faces are turned upside down reflecting the lack of interference from integration across the whole face when inverted.

A further method of assessing configurational/ holistic face processing is the composite task (Figure 3) (Young et al., 1987). In this task participants are asked to match only the top or bottom half of a face between stimuli. The pairs to be matched are then either aligned or misaligned with different identities presented in the irrelevant half of the face. It is found that matching half a face is much more accurate when it is not aligned with the rest of the face compared to when alignment with the irrelevant section creates a whole face. In the latter case the participant seems unable to avoid processing the irrelevant section of the face. This suggests a mandatory recruitment of a more global representation of the face, which in this task acts to weaken performance. Using the face composite task, Nishimura et al. (2008) found that both adults with ASD and control participants were more accurate when the faces were misaligned. There was no difference between the groups when the task involved matching the top or the bottom half of the face. A similar study has used a congruency manipulation of the composite task (Gauthier et al., 2009). In this manipulation, the stimuli are either the whole target face or a distracter identity (congruent trials) or the target identity is cut across the center of the image and shared between the two stimuli presented (incongruent trials). Gauthier et al. (2009) found that adolescents with autism performed more poorly overall when matching faces, and where control participants showed no difference between congruent and incongruent conditions when the faces were misaligned, the ASD participants demonstrated a disadvantage when matching the misaligned face in the incongruent compared to the congruent condition. This suggests that even when the faces were misaligned, the ASD participants were not able to discount the irrelevant section of the face. Although this result seems difficult to interpret, it is interesting to note that this effect is most pronounced when participants with ASD are asked to make judgments about the bottom half of the face, suggesting that they are having trouble ignoring the matching top half of the distractor face. The authors suggest that unlike controls, ASD participants are using holistic processing in the misaligned condition, however, this result perhaps points more to an inability to direct attention appropriately. In this task the part of the face required to successfully carry the task was well-cued and as such this result is difficult to assimilate into the larger body of evidence.

**Figure 4:**
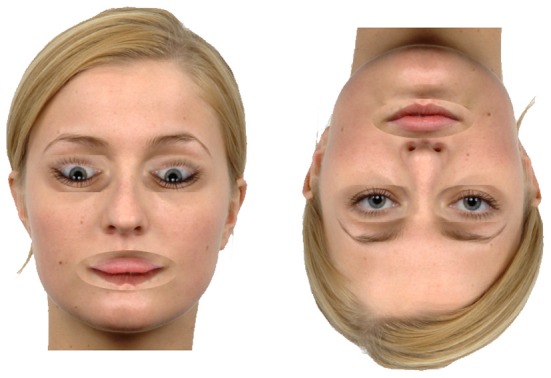
**The Thatcher illusion.** Both faces have the eyes and mouth rotated “in place,” however, only the upright face appears grotesque. Unedited face not shown (Langner et al., 2010).

The Thatcher illusion (Thompson, 1980), involving inverting the eyes and mouth in place within an upright face, also manipulates the spatial relationships between features of a face to create an effect that is easily apparent in an upright face but much more difficult to see when the face is inverted (Figure 4). This difficulty suggests that the manipulation rests on the relationship between the features of the face rather than the features themselves (Bartlett and Searcy, 1993). Both Riby et al. (2008) and Rouse et al. (2004) have shown that autistic children also find it more difficult to identify Thatcherisation (assessed by a two alternative “oddness” task) in inverted faces. Both autistic and control groups were extremely accurate in the upright condition and their performance dropped dramatically in the inverted condition, suggesting that both groups process the upright face holistically/configurationally.

Despite the difficulty in comparing research within a population that can vary so markedly in symptomology, age, functioning and even face recognition ability, it is quite clear from these studies that, on average, groups of individuals with a diagnosis within the autism spectrum are generally less accurate at carrying out the tasks outlined above. Yet they almost always demonstrate a configurational processing strategy as measured via an inversion effect. An extremely comprehensive review of the face processing research in autism has recently been carried out by (Weigelt et al., 2012). Alongside the studies mentioned here this review also addresses the results of studies that include a memory component or provide a less clear cut test of configurational/holistic processing. Yet the authors arrive at the same conclusion that participants with autism display a face processing style that is qualitatively similar to controls in terms of global processing when no memory component is involved in the task.

Moreover, most of the studies described did not cue the participants to consciously employ a part-based or holistic strategy. Therefore, it can be concluded that people with autism or autism spectrum disorder are able to and likely do employ a holistic/configurational strategy preferentially. This indicates that a lack of holistic/configurational processing may not be the leading cause of poor face recognition. It is interesting that this data strongly hints at participants using a different, or a suboptimal configurational strategy involving the mouth region and perhaps with an altered use of the eye region, as opposed to a part-based strategy. This is in line with findings from eye tracking studies that many people with autism place much less emphasis on the eye regions (e.g., Klin et al., 2002; Dalton et al., 2005), or show an altered scanning strategy when looking at faces (e.g., Wilson et al., 2012). Whether or not people with autism actively avoid eye contact or fail to appreciate the importance of the eye region (for discussion of this issue see Itier and Batty, 2009) what the data explored here shows is that not paying attention to the same regions of the face as a control population cannot be taken as general evidence of a part based face recognition strategy.

Additionally, it is important to consider that autism is a developmental disorder. There is debate as to whether face recognition deficits in autism are an inherent feature of an overall local processing bias or whether it is due to an insufficient level of expertise caused by a lack of intrinsic reward gained from social interaction (Hobson et al., 1988). As avoidance of social interaction, including with faces, is central to autism it is clear that developmental experience with faces will be different/lacking. Those who have spent less time interacting with people socially will have developed relatively less expertise in face recognition. The results discussed here would suggest that holistic/configurational processing is a hallmark of face recognition even with reduced expertise (for a discussion of the role of expertise in face recognition see McKone and Robbins, 2011). Yet expertise and motivational factors are surely going to play a large part in developing an optimal representation strategy. When it comes to stimuli that are as similar as each other as faces, yet convey so much important information, a lack of avoidance of the eyes in particular (compared to other features) and a willingness to engage with others socially may together be the driving factor behind development of an optimally tuned and elaborated holistic representation, rather than development of a holistic representation itself.

## Face processing in schizophrenia

Compared to the early onset of autism, schizophrenia onsets in late adolescence, well after considerable expertise in recognizing faces has been developed. Yet in schizophrenia a relative over processing of the local features of stimuli is also a hallmark (Braff, 1993), and a general deficit in face recognition is observed (Marwick and Hall, 2008). It is important to note that a large body of literature has shown that people with schizophrenia have deficits in the recognition of emotion yet the literature on identity recognition is smaller and more varied in its findings (Marwick and Hall, 2008). None the less, there is a very strong suggestion of face identity processing being generally altered in schizophrenia. For example, Chen et al. (2009) have shown that patients show reduced face detection and discrimination ability, particularly with a working memory load component, but also without. Crucially, they have shown that this deficit is independent of deficits in the recognition of other objects. Suggesting that face recognition performance is degraded via a different mechanism to that responsible for recognition of other objects.

Assessing holistic face recognition in schizophrenia, Butler et al. (2008b) have tested the face inversion effect by comparing matching of sequentially presented faces and houses presented either upright or inverted. They initially adjusted the duration of the stimulus presentation so that upright houses could be matched with 70% accuracy or above by all participants. Patients needed ~100 ms longer than controls to reach this standard. This manipulation is quite important as it shows that the results are not due to factors such as an overall lack of attention to the task in one participant group. Despite this matching, they found that participants with schizophrenia were less accurate at matching upright faces and their relative drop in accuracy when the face was turned upside down was just as great as the controls. In other words they showed a standard face inversion effect. In the same study Butler et al. (2008b) also carried out a configuration/ feature manipulation test with upright and inverted faces. The size of the inversion effect did not differ between groups regardless of whether the participants were carrying out the matching task with stimuli that differed featurally (by having the shape of the eyes or mouth manipulated), or configurationally (by having the location of facial features changed). This suggests that both a featural change and a configurational change are affected by inversion of the face to the same extent whether patient or control. If a part-based strategy were apparent this should not be the case.

In a similar design Shin et al. (2008) assessed accuracy for matching of simultaneously presented faces. They did not adjust the duration of the faces between participant groups yet their design in this regard could be viewed as being very similar to Butler et al. (2008b). Shin et al. (2008) used chairs as a comparison stimulus and found equal upright performance for both groups on the chair stimuli. They found that patients were most impaired at matching faces within which the features (eyes or mouth) had been moved. In fact, the patients were at chance in this condition leaving no room to assess whether an inversion effect occurred. This manipulation most affects configurational information so drastically reduced performance is informative given comparable performance with non-face stimuli. The patient group were also impaired (though not at chance) when matching faces within which the features themselves had been changed. This was less pronounced, however, and in this condition the inversion effect was as large as the control group. As this manipulation also changes the configuration of the face this can be taken as evidence of configurational/holistic processing. A further study using the same stimuli assessed people with schizophrenia and people at ultra-high risk of developing schizophrenia (Kim et al., 2010). Although carried out by the same research group this study recruited participants who had not completed the previous study and found similar results. They found that the patient group were impaired at discriminating between upright faces with the features moved within the face and were very nearly at chance. Given this result this inversion effect cannot be adequately measured. The patient group were also impaired at discriminating both faces and chairs with the features swapped for those of another identity/chair. Despite being a feautral manipulation this condition also changes the configuration of the face so it is significant that in both of these conditions the size of the inversion effect did not differ from controls. The ultra-high risk group were not, on average, at chance in the upright-face features-moved condition, however, looking at the data presented, a cluster of participants appear to have been and as such this makes interpretation of the inversion data difficult. Interestingly, this group of participants were not impaired at matching upright faces within which the features had been swapped. This overall pattern of results suggests that diagnosed patients' impairment is due to impairment in both configurational and part-based processing, or some other factor, as a deficit in configurational/holistic processing alone should result in a pattern of results resembling the ultra-high risk populations. The lack of impairment in the ultra-high risk group in the feature change condition does imply that the impairment in face matching is an effect that develops in stages, with the ability to use second order relational information becoming less reliable prior to a decline in ability at matching featural or part-based information.

Recently, Soria Bauser et al. (2012) have carried out an inversion study where they have assessed patient's performance using a range of standard face recognition tests and a match to sample task involving upright and inverted faces, bodies and cars. In all aspects of performance the patients were impaired compared to controls. In particular, patients performed the upright match to sample task less accurately and with longer reaction times across all stimulus categories. They also showed an inversion effect for all stimulus categories with only the difference in reaction time showing a reduced inversion effect.

Other methods for investigating configurational/holistic processing that include more varied aspects of face processing also draw a similar conclusion. For example, Schwartz et al. (2002) have used similar inversion paradigms to the studies above but included a memory aspect in the task. They have shown that patients do indeed show an inversion effect and a composite effect similar to that of controls when recognizing previously learned faces.

The results found by Butler et al. (2008b) and Shin et al. (2008) are particularly important because performance with the non-face stimuli was equal between groups. This shows that impairments measured are due to altered face recognition abilities rather than a more general visual deficit and yet the patients displayed the same pattern of results as controls; they were just worse overall. Therefore, it seems that it is not only (if at all) a lack configurational/holistic processing that is driving the reduced face recognition ability in patients. The study by Kim et al. (2010) offers some insight in that perhaps there is a stage when a specific configurational/holistic processing deficit is a key feature in schizophrenia, however, with progression of the disorder this impairment becomes more encompassing.

## Conclusion

On the whole, even when large group level impairments in face processing are apparent, the results from participants with autism, ASD or schizophrenia do not show a strong suggestion of a selective lack of holistic/configurational face processing. Rather they seem to show a more pervasive processing problem that could involve an inefficient representation of the face whether this be a holistic, configurational, or part-based representation.

This is important because it raises some interesting questions about our understanding of face processing. Both autism and schizophrenia are strongly associated with a predominant bias toward relying on localized or fragmented information in visual processing. When you consider that both groups have measurable face recognition deficits and show strong signs of configurational/holistic processing of faces this suggests that two options are possible. The first is that our tests do not adequately isolate and measure configurational/holistic processing or that configurational/ holistic processing is such a key strategy for recognizing faces that it is necessary and engaged even in disorders of holistic processing such as autism.

Do our tests adequately measure holistic/configurational processing? This is a question that is hotly debated in the literature (e.g., McKone et al., 2013), however, I feel that it is not necessary to look at the tests in detail to address this question. It is only necessary to note that the literature has not yet satisfactorily addressed what it means in neural terms to carry out holistic or configurational processing as opposed to part-based processing. This shows that we have not yet found exactly the right way to conceptualize how face recognition occurs. Both groups of people considered here, those with a developmental disorder and those with a disorder that onsets in late adolescence, show selectively impaired or altered face recognition yet also show key patterns of data suggestive of configurational/holistic processing. Because of this we can definitely say that we have not yet identified a key aspect of successful face recognition. We have spent a considerable amount of research time investigating configurtional/holistic processing of faces and our tasks for measuring these essentially are our definition of what we mean when we talk about configurational or holistic processing. We can therefore conclude already that the key aspect we have not yet found will not clearly fit within a part-based and configurational/holistic processing dichotomy.

Moving on to the second suggestion; can it be the case that both populations display automatic configurational processing despite a local processing bias in everything else? I believe that it is possible and in regards to the strength of the local bias (particularly in autism) this could be seen as testament to the “specialness” of faces. That faces prompt this strategy to be applied naturally by populations who are not logically expected to do so shows the strength of the inclination to apply this strategy to face stimuli. This is even more strongly highlighted by the autistic populations who may, in addition to a local processing bias, be less motivated from a very early age to engage with faces. This would result in less motivation to build up the expertise to develop a fully realized database of holistic representations of faces (Hobson et al., 1988). The results presented cannot resolve the debate about whether the specialness of faces is in any way genetic, or whether it can be fully realized through experience (Morton and Johnson, 1991). It does, however, suggest that what we consider to be configurational/holistic processing does occur even in populations for whom logic currently suggests it should not and therefore is a necessary and somewhat unique aspect of face recognition.

If configurational processing is necessary yet simply applying it is not sufficient for optimal face recognition, where should we be looking now? I believe that autism and schizophrenia can offer some insight here in the form of the models that have been proposed to describe the disorders. Those mentioned in the opening of this article (e.g., Frith, 2004; Happé and Frith, 2006; Krishnan et al., 2011) and other similar models (e.g., Phillips and Silverstein, 2003) offer insight into the kind of neural connectivity and computation that needs to be running smoothly for complicated visual tasks like face recognition to be carried out optimally. They highlight the importance of a whole network of areas of the brain communicating effectively with each other to carry out the millisecond by millisecond perceptual processing we are engaging in. What studies of these patient populations show us is that the progress we have made using the modular view of brain function as it relates to face recognition needs now to be enhanced by bringing back into the modular framework an understanding of how the networks of various sizes throughout the brain act in concert to provide what is necessary for the complex behaviors we are researching.

Those who proposed the configurational and holistic accounts of face recognition acknowledge that the nameable parts of a face are also an important perceptual aspect of a face (e.g., Rhodes, 1988). Indeed, a holistic representation of a face could not be achieved without some part-based processing occurring. This is because our understanding of visual processing as a hierarchy of stages makes it mandatory that the visual input will be initially decomposed into more basic elements, such as oriented lines, to then be recombined into the meaningful units at various spatial scales that convey the face of a particular person. To understand optimal face recognition we will need to understand which aspects of the face are necessary to be included in the feed forward sweep of processing and how these aspects are identified from the decomposed input coming from the first stages of the visual hierarchy. We'll also need to understand the parameters within which timing of the transfer of information must occur to ensure that the right information reaches crucial stages of higher level processing. Additionally, understanding the role of feedback in subsequently supplementing the detail passed forward in the first pass of processing. Each of these questions is beginning to be effectively addressed via electrophysiological studies in macaques and the results very much highlight the physically and temporally distributed nature of face recognition. For example, Freiwald and Tsao (2010) have demonstrated that distinct face selective areas spanning the macaque temporal lobe can be localized using fMRI. Subsequently investigating the selectivity profiles of cells in these areas they show that each area has a distinct pattern of selectivity for viewpoint and identity. The selectivity and temporal response profile suggest a sequential (as opposed to parallel) anterior flow of information through the regions. In addition to this, Sugase-Miyamoto et al. (2011) highlight the temporal evolution of face selective neurons' responses to face stimuli. They suggest that a global snapshot of the face is first processed and then filled in with the detail necessary for the judgment at hand via inter-area connections, recurrent contributions or feedback. Although there is also a suggestion that no contribution of recurrent processing or feedback need be involved (Riesenhuber and Poggio, 1999) there is still the necessity for the stimulus to pass through many areas of the visual hierarchy and co-ordination of the sweep of information remains crucial to this feed forward sweep containing the optimal configurational information. These questions sit comfortably within network models and as we address these questions in autism, schizophrenia and face recognition we can look forward to the opportunity to transfer across research questions the techniques and knowledge developed.

To conclude, studies addressing the presence of configurational/ holistic processing of faces in people with autism and schizophrenia suggest that these groups do engage a configurational/holistic processing style. This is despite an apparent emphasis on a part-based strategy when carrying out other tasks. This hints at the use of holistic/configurational processing as necessary, but simply applying it as a strategy is not sufficient for optimal face recognition. Autistic children in particular may be relying on sub optimal configurational cues or a holistic template that involves a greater reliance on the mouth region. This understanding of configurational/holistic processing suggests that to better understand how faces can be represented within the brain we must now look to elaborate modular understanding of face recognition and concentrate on the key aspects of the network involved in optimal face recognition.

### Conflict of interest statement

The author declares that the research was conducted in the absence of any commercial or financial relationships that could be construed as a potential conflict of interest.

## Footnotes

^1^It's notable that these descriptions are based on the DSM-IV-TR (American Psychiatric Association, 2000) however the DSM-V (American Psychiatric Association, 2013) has been released at the time of writing. As there has been no time yet for any research based on the DSM-V criteria for diagnosis this description is based on the DSM-IV-TR.
